# Disruption of multiple copies of the Prostaglandin F2alpha synthase gene affects oxidative stress response and infectivity in *Trypanosoma cruzi*

**DOI:** 10.1371/journal.pntd.0010845

**Published:** 2022-10-19

**Authors:** Ana Maria Murta Santi, Juliana Martins Ribeiro, João Luís Reis-Cunha, Gabriela de Assis Burle-Caldas, Isabella Fernandes Martins Santos, Paula Alves Silva, Daniela de Melo Resende, Daniella Castanheira Bartholomeu, Santuza Maria Ribeiro Teixeira, Silvane Maria Fonseca Murta

**Affiliations:** 1 Grupo Genômica Funcional de Parasitos, Instituto René Rachou, Fiocruz Minas, Belo Horizonte, Minas Gerais, Brazil; 2 Departamento de Parasitologia, ICB, Universidade Federal de Minas Gerais, Belo Horizonte, Minas Gerais, Brazil; 3 Departamento de Medicina Veterinária Preventiva, Escola de Veterinária, Universidade Federal de Minas Gerais, Belo Horizonte, Minas Gerais, Brazil; 4 Departamento de Bioquímica e Imunologia, Universidade Federal de Minas Gerais, Belo Horizonte, Minas Gerais, Brazil; INGEBI, ARGENTINA

## Abstract

Chagas disease, caused by the protozoan *Trypanosoma cruzi*, is a serious chronic parasitic disease, currently treated with Nifurtimox (NFX) and Benznidazole (BZ). In addition to high toxicity, these drugs have low healing efficacy, especially in the chronic phase of the disease. The existence of drug-resistant *T*. *cruzi* strains and the occurrence of cross-resistance between BZ and NFX have also been described. In this context, it is urgent to study the metabolism of these drugs in *T*. *cruzi*, to better understand the mechanisms of resistance. Prostaglandin F2α synthase (PGFS) is an enzyme that has been correlated with parasite resistance to BZ, but the mechanism by which resistance occurs is still unclear. Our results show that the genome of the CL Brener clone of *T*. *cruzi*, contains five PGFS sequences and three potential pseudogenes. Using CRISPR/Cas9 we generated knockout cell lines in which all PGFS sequences were disrupted, as shown by PCR and western blotting analyses. The PGFS deletion did not alter the growth of the parasites or their susceptibility to BZ and NFX when compared to wild-type (WT) parasites. Interestingly, NTR-1 transcripts were shown to be upregulated in ΔPGFS mutants. Furthermore, the ΔPGFS parasites were 1.6 to 1.7-fold less tolerant to oxidative stress generated by menadione, presented lower levels of lipid bodies than the control parasites during the stationary phase, and were less infective than control parasites.

## Author summary

Prostaglandin F2α synthase (PGFS) has been associated with *T*. *cruzi* resistance to benznidazole (BZ), but the real involvement of this enzyme in the resistance phenotype is still uncertain since different studies in the literature point in different directions. Here we demonstrated that the deletion of all copies of the PGFS gene in *T*. *cruzi* does not affect the parasite’s resistance to BZ or nifurtimox (NFX), but results in reduced tolerance to oxidative stress caused by menadione. The PGFS knockout mutants are less infective, and, at the stationary phase, the parasites have fewer lipid bodies than the control parasites. Thus, our results suggest that this enzyme appears to have a regulatory role in defence against oxidative stress and parasite infectivity.

## Introduction

*Trypanosoma cruzi* is the causative agent of Chagas disease, transmitted by Hemiptera insects of the subfamily Triatominae [1]. Nonvectorial transmission also occurs by blood transfusion, congenital transmission, organ transplantation, and consumption of food contaminated with infectious stages of the parasite. It is currently estimated that 6 to 7 million people are infected, with another 75 million at risk of infection [2]. Chagas disease is endemic in 21 Latin American countries and due to increased migration, the disease has spread across Europe, Australia, Japan, Canada, and the southern United States [3].

There is no vaccine against Chagas disease and only two drugs are used for the treatment: benznidazole (2-nitroimidazole; BZ) and nifurtimox (5-nitrofuran; NFX). Both compounds produce undesirable side effects and present low cure rates mainly in the chronic phase of the disease [4]. However, BZ treatment for the chronic phase of the disease may improve immune response and myocardial function [5]. Differences in susceptibility to BZ and NFX between *T*. *cruzi* strains and/or the genetic diversity of the host might explain, in part, the variations in the efficacies of these trypanocidal drugs [4]. Recently, dormant, non-proliferating amastigotes have also been implicated in drug resistance [6].

Both BZ and NFX are nitroheterocyclic compounds that act as pro-drugs. Their activation by nitroreductases results in the formation of reactive oxygen species, which are extremely toxic to the parasites since they bind to DNA, RNA, lipids, proteins, and low molecular weight thiols [7–10]. In *T*. *cruzi*, BZ is also responsible for oxidizing the nucleotide pool, which is later incorporated by DNA polymerases, generating mutations [11]. One of the main resistance mechanisms developed by the parasite has been linked to the inhibition of the bioactivation of BZ and NFX by nitroreductases like, for example, nitroreductase NTR-1 [7,8,12–14].

It was also observed that prostaglandin F2 synthase (PGFS), commonly known as Old Yellow Enzyme (OYE), is linked to *T*. *cruzi* drug resistance. This enzyme uses NAD(P)H to catalyse the reduction of 9,11-endoperoxide PGH_2_ to prostaglandin F_2_α (PGF_2_α) and hydrogen peroxide. It has been shown that PGF_2_α is released by *T*. *cruzi* and it has been proposed that the liberation of this eicosanoid could be related to the survival of the parasites in the host, but its real role has not yet been fully elucidated [15–17]. PGFS can also catalyse the reduction of certain drugs, and it has been demonstrated that it can reduce NFX under anaerobic conditions, but not BZ [18,19]. The deletion of copies of the PGFS gene in *T*. *cruzi*, low transcription levels of this enzyme [20], and a decrease in PGFS protein expression [21] are all associated with in vitro-induced BZ resistance in this parasite. In *T*. *cruzi* with natural resistance to BZ, transcripts of the PGFS gene were likewise found to be downregulated [22]. It has also shown that parasites overexpressing PGFS are more susceptible to BZ and NFX [16,23]. On the other hand, another study demonstrated that an *in vitro*-induced BZ-resistant *T*. *cruzi* strain, which lacks NTR-1 enzyme, had increased PGFS transcripts levels [14]. Another study showed that, among five different populations of *T*. *cruzi* with *in vitro* induced resistance to BZ, one showed increased PGFS expression [13].

To better understand the real role of PGFS in *T*. *cruzi* BZ resistance, we used CRISPR/Cas9 to knockout all PGFS genes in the CL Brener clone and then assessed the mutant’s phenotype in terms of susceptibility to BZ, NFX, and oxidative stress induced by menadione. We also investigated the NTR-1 transcript levels in the mutants, their infectivity, and the amount of lipid bodies.

## Methods

### Parasites and cultivation

Epimastigote forms of the CL Brener strain of *T*. *cruzi* were grown at 27°C in LIT medium supplemented with 10% inactivated fetal bovine serum as previously described [24]. Cultures were maintained by performing a weekly passage, inoculating 2 x 10^6^ parasites for each 5 mL of medium. All experiments were performed using epimastigotes in the logarithmic growth phase.

### PGFS copy number analysis

To evaluate the copy number and sequence variability of PGFS in CL Brener, the nucleotide sequence of the gene TcCLB.508461.80 was retrieved from TriTrypDB 36 and used in a BLASTn against the CL Brener genome, assembled with a combination of SMRT long-reads, Illumina and Sanger reads. Such genome data has not been published yet (DC Bartholomeu, personal communication). In the BLASTn analysis [25], the low complexity filter was turned off and BLASTn matches were filtered by a minimal identity of 75% and minimum query coverage of 75%. Since the TcCLB.508461.80 gene has 1,140 nucleotides, minimal matches of 855 nucleotides were accepted. The BEDTools getfasta program [26] was used to extract the nucleotide sequences from the matches. To search for internal stop codons, the sequences were translated using the Expasy Translate Tool [27] and then aligned using MAFFT [28]. To estimate the phylogeny of the PGFS family within *T*. *cruzi* clade, the nucleotide sequence of all ortholog genes for the CL Brener TcCLB.508461.80 PGFS gene, present in any *T*. *cruzi* strain deposited in TriTrypDB 36, were retrieved, and combined with the genes identified in the CL Brener long-read assembly (DC Bartholomeu, personal communication), totalizing 91 genes (S1 Table). Then, genes that had more than 700 nucleotides were aligned using MAFFT, and the genes TcYC6_0070500-RA, C3747_1g202-t42_1, TcBrA4_0060950-RA, TcBrA4_0070060-RA, TcYC6_0070480-RA, TcBrA4_0065060-RA, TcBrA4_0070120-RA, and C4B63_396g4-t42_1 were manually removed, as they were lacking regions or presented non-ACTG nucleotides. This resulted in a total of 67 genes to be analyzed (S2 Table). Finally, the alignment was truncated in position 783, to keep only regions that were present in all evaluated genes. A Bayesian phylogeny analysis was performed with this alignment, using MrBayes [29], with 3,000,000 MCMC generations, and the HKY nucleotide substitution model with the variation following a gamma distribution, as selected by the ModelTest-NG [30] program. Finally, the tree was generated using ITOL online tool [31].

### PGFS knockout

Disruption of PGFS genes by CRISPR/Cas9 was performed as previously described by Burle-Caldas et al, 2018 [32]. The sgRNAs were selected using the Eukaryotic Pathogen CRISPR guide RNA/DNA design tool (EuPaGDT) [33] based on the TcCLB.508461.80 sequence. DNA templates to produce sgRNAs were obtained by PCR and the sgRNAs were *in vitro* transcribed from the DNA templates using the MEGAshortscript T7 Transcription Kit (Thermo Fischer Scientific, USA) according to the manufacturer’s instructions, and purified by the phenol-chloroform method. The parasites constitutively expressing the endonuclease SpCas9 were transfected with a sgRNA transcribed *in vitro* and a donor DNA. The donor DNA contains stop codons in three different reading frames to disrupt the PGFS coding sequence and the XhoI restriction enzyme recognition site. For homologous recombination at the desired location, 30 nucleotides were added to each end of the donor (S3 Table).

Transfections were performed using the Amaxa Nucleofactor (Lonza) X-001 program. Parasites (4 x 10^7^ epimastigotes) were transfected with equimolar amounts of sgRNA and donor DNA. To delete all copies of PGFS present in the CL Brener strain, the parasites were re-transfected 4 times, with intervals of approximately two weeks between each transfection. After the last electroporation, the parasites were cloned by limiting dilution in a 96-well plate.

For screening the clones, the genomic DNA of WT and mutant parasites was extracted by the phenol/chloroform method. The complete PGFS coding sequence was amplified by PCR and the PCR products were purified using the QIAquick PCR Purification Kit (Qiagen). After purification, the fragments were digested with the XhoI restriction enzyme to evaluate if all PGFS alleles have been correctly edited.

### Add-back parasites

To generate the add-back parasites, the PGFS coding sequence was cloned into the pROCK_HYG vector using the Gibson Assembly Reaction (NEB) and primers designed at NEBuilder (https://nebuilder.neb.com/) (S3 Table). After ligation, plasmids were incubated with *E*. *coli* TOP10F’ bacteria (Invitrogen) at 4°C for 30 min and then at 42°C for 45 s in a dry bath. Subsequently, the bacteria were incubated at 37°C for 1 h to express the gene for ampicillin resistance and then plated on a solid LB medium in the presence of ampicillin (Sigma). To evaluate the sequences, plasmids obtained from different clones were sequenced by the Sanger method on the Instituto René Rachou FIOCRUZ/MG sequencing platform. Sequence analyzes and contigs assembly were performed with the aid of DNAstar (Lasergene) and Multalin (http://multalin.toulouse.inra.fr/multalin/) softwares. After confirming the correct PGFS sequence, 100μg of plasmid pROCK_HYG_PGFS was linearized with the restriction enzyme NotI (Promega) and precipitated with sodium acetate and isopropanol. Transfection of the epimastigote forms of the ΔPGFS mutants was performed as described in DaRocha et al., 2004 [34], and the parasites were selected using 200 μg/ml of hygromycin (Invitrogen).

### Western blot

Western blot assays were performed to evaluate PGFS expression in the epimastigote forms of WT, Cas9 expressing parasites, KO mutants and add-back parasites. The rabbit polyclonal antibody anti-TcPGFS [20] was used at a concentration of 1:500. Anti-α-tubulin antibody at a concentration of 1: 5,000 was used as a normaliser in the densitometric analysis to compare all the parasites to the WT.

### Growth curve of epimastigote forms

To evaluate the growth of the parasites, epimastigote forms of *T*. *cruzi* (2 x 10^6^ parasites/mL) were inoculated in LIT medium, and the parasite number was determined daily by using the cell counter Z1 Coulter Particle Counter (Beckman Coulter).

### IC_50_ assays

To test parasite susceptibility, 2 x 10^6^ replicative and non-infective epimastigote forms were incubated in 1 mL of LIT medium containing different drug concentrations ranging from 1.25 to 15 μM of BZ, 0.3125 to 5 μM of NFX, and 1.0 to 4 μM of menadione. After 7 days, the number of parasites grown in the absence and presence of each drug was determined by using the Z1 Coulter Particle Counter (Beckman Coulter). The 50% growth inhibitory concentration (IC_50_) was determined using the non-linear regression–variable slope model as per the equation "log (inhibitor) vs. response" in GraphPad Prism v.8.2.0.

### RT-qPCR

Epimastigote forms of *T*. *cruzi* (approximately 10^8^ cells) were harvested and resuspended in 1 mL TRIzol Reagent (Invitrogen) and total RNA was extracted using the chloroform method. After treating the RNA with DNase I (Ambion), the cDNA was produced using Invitrogen’s Superscript II reverse transcriptase according to the manufacturer’s instructions. All cDNA samples were diluted to 100 ng/μL and used in the RT-qPCR amplification reaction, which was performed using 1X SYBR GREEN master mix (Applied Biosystems) and the specific primers listed in S3 Table. The housekeeping gene hypoxanthine-guanine phosphoribosyltransferase (HGPRT) was used as a normaliser. Amplifications were performed using a QuantStudio 12 K Flex system (Thermo Fisher Scientific). The PCR conditions were as follows: 95°C for 10 min, 40 cycles of denaturation at 95°C for 15 seconds, and the annealing/extension at 60°C for 1 min. Fluorescence levels were measured after each extension step. The fold-change was calculated using the comparative CT method (2^−ΔΔCT^ Method).

### Nile Red staining and quantification on epimastigote forms

*T*. *cruzi* epimastigotes (3 x 10^6^ cells) were washed twice with PBS and incubated in 1.5 μg/mL Nile Red (Thermo Fischer Scientific, USA) for 30 minutes at room temperature and protected from light. To perform the confocal microscopy, an aliquot of each cell suspension was attached to glass coverslips coated with 0.1% poly-L-lysine for 10 minutes and after, fixed in 4% formaldehyde for additional 10 minutes at room temperature and protected from light. The glass coverslips were washed thrice with PBS and incubated with *ProLong* Gold antifade reagent and the images were obtained using the Nikon C2+ Confocal Microscope and analyzed using the NIS Elements Microscope Imaging Software (Nikon). For the evaluation the amount of lipid bodies in the parasites, a total of 30,000 events/sample were acquired in the BD FACSCalibur flow cytometer, and the data obtained were analyzed using the Flow Jo software (Flow Cytometry Analysis Software, version 10) [35].

### L929 fibroblast infection

These experiments were performed using Cas9 expressing parasites, KO mutants and add-back parasites. To obtain the trypomastigotes, L929 fibroblasts were infected with an aged parasite culture (15 days after the stationary phase was achieved). After 24h incubation, washing was done with PBS to remove the parasites that did not infect the fibroblasts, and a RPMI medium with 10% horse serum was added to kill the epimastigote forms. After 24h, the medium with horse serum was removed and RPMI with fetal bovine serum was added. After ten days, the bottles were transferred to an incubator at 33°C to release the trypomastigote forms in the supernatant. For the *in vitro* infection experiment, L929 fibroblasts were counted and plated in 24-well plates containing glass coverslips (20,000 cells per well). After 24 hours, the fibroblasts were infected with trypomastigote forms recovered from the supernatant (10 parasites per fibroblast) for 2 hours. The parasites that were not able to infect the macrophages were removed by successive washings with PBS and the infected macrophages were incubated in RPMI-1640 medium. The infectivity of the parasites was evaluated after 48h. The slides were stained with Rapid Panoptic (Laborclin), photographed, and the infection was quantified by counting intracellular amastigotes using the free ImageJ program.

### Statistical analysis

For all experiments, at least three technical replicates were performed for each of the three biological replicates. Data were analyzed using GraphPad Prism v.8.2.0. Ordinary one-way ANOVA or two-way ANOVA test with Bonferroni post hoc test were used to compare WT and mutant parasites. Statistical significance was set at *p* < 0.05. The *p*-values were reported as per the GraphPad Prism format, where ns (*p* > 0.05), * (*p* ≤ 0.05), ** (*p* ≤ 0.01), *** (*p* ≤ 0.001), and **** (*p* ≤ 0.0001).

## Results

### PGFS is a multiple copy gene in *T*. *cruzi*

The complete gene repertoire encoding PGFS in the CL Brener clone of *T*. *cruzi* was evaluated after analysing an improved version of this genome which was assembled using a combination of long PacBio reads, Illumina and Sanger reads [36,37]. We identified a total of 8 sequences encoding PGFS, 6 in scaffold 67 and 2 in scaffold 34 (S2 Table), which appears to be in tandem in each scaffold (GenBank: ON567257—ON567264). This is an improvement to what is currently annotated in the CL Brener assembly, available in TriTrypDB 36, in which only three sequences of PGSF gene were identified: TcCLB.508461.80 for CL Brener Non-Esmeraldo-like and TcCLB.506147.9 and TcCLB.507617.9 for CL Brener Esmeraldo-like. The genome of the CL Brener clone is a hybrid genome derived from two distinct parasite lineages and the annotated dataset indicates for each gene its haplotype assignment (i.e. "Esmeraldo-like" or "non-Esmeraldo-like").

The MAFFT alignment of the 8 retrieved sequences demonstrated that they share a large part of their sequences, presenting a few SNPs and some insertions and deletions (S1 Fig). Sequence translated using the Expasy Translate Tool showed that 3 have internal stop codons and were considered as pseudogenes (S2 Fig).

**Fig 1:**
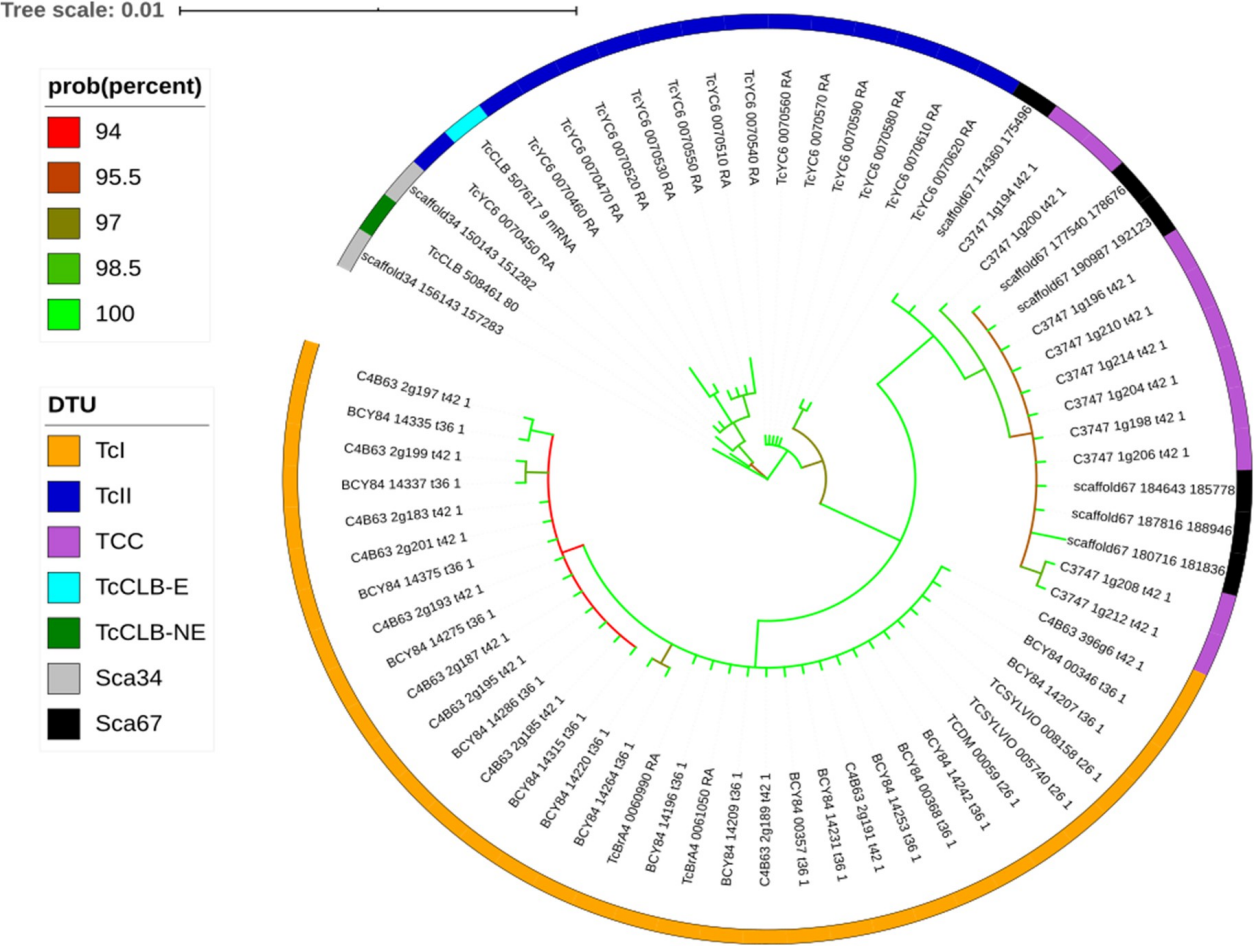
Bayesian phylogram inferred from PGFS sequences from different *T*. *cruzi* strains.

A Bayesian phylogeny analysis was performed by comparing all the copies found in CL Brener genome with the genes of other *T*. *cruzi* isolates, from the DTUs TcI, TcII, and TcVI. The results show that the scaffold 34 genes clustered with the TcVI CL Brener sequences from TriTrypDB 36 (TcCLB.508461.80 and TcCLB.507617.9) and with Y strain genes, which belong to TcII. On the other hand, scaffold 67 genes clustered with genes from the TCC (DTU TcVI) genome, which was assembled using SMRT-long-reads [36] (Fig 1). These results reinforce the importance of using SMRT-long reads to assemble repetitive multigene family regions, as genes close to the TCC cluster were not observed in the CL Brener genome currently available in the TriTrypDB 36.

### PGFS is not an essential gene for *T*. *cruzi* epimastigotes

**Fig 2:**
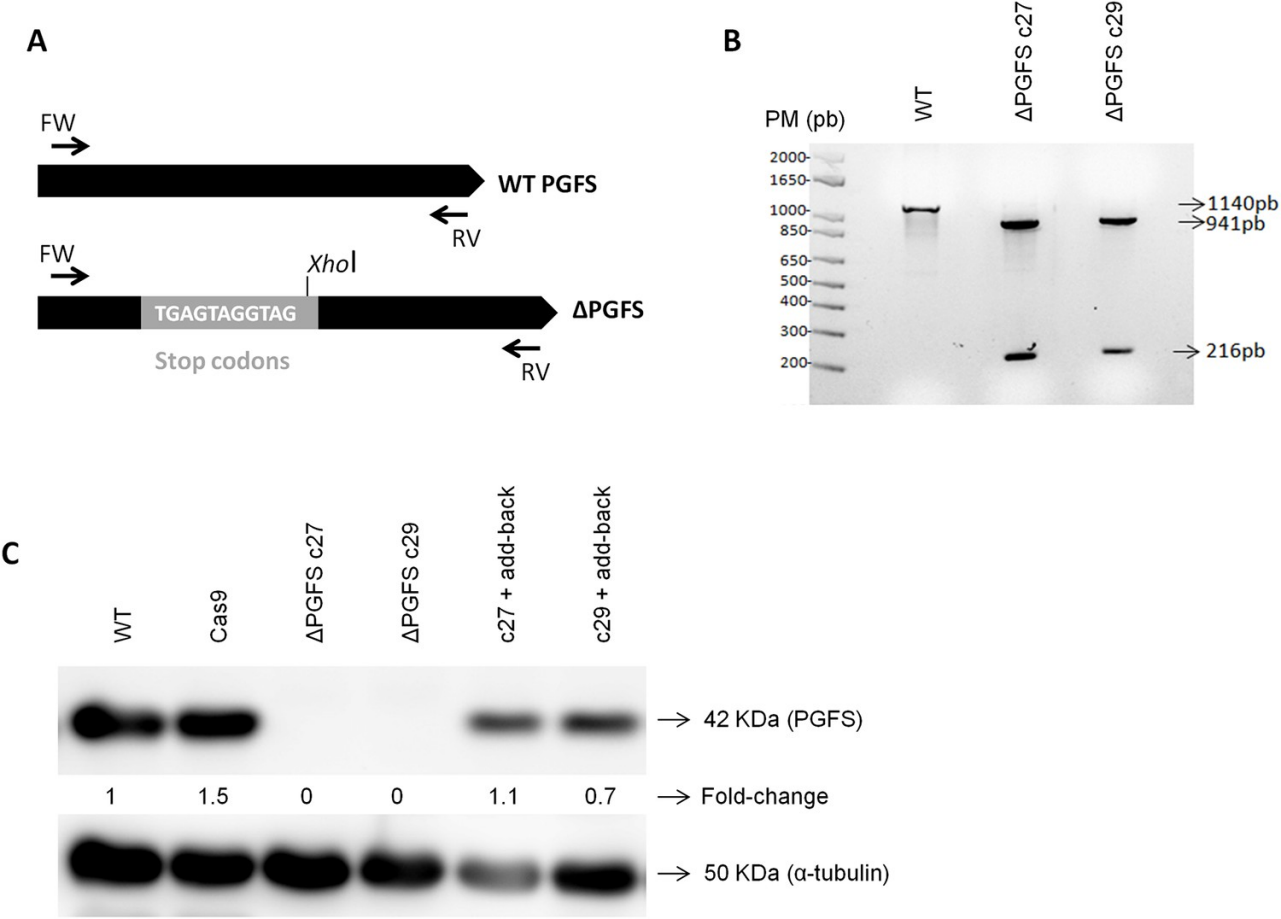
Characterization of *T*. *cruzi* ΔPGFS mutant clones and PGFS add-backs. Screening of *T*. *cruzi* PGFS knockout clones after CRISPR/Cas9 with sgRNA187 was performed by both PCR and western blot. (**A)** Schematic representation of PGFS sequence before and after the insertion of donor DNAs with stop codons and XhoI restriction site; (**B)** The complete PGFS coding sequences (1140 bp) were amplified by PCR. The primers can successfully amplify both the WT sequence and the mutant sequences containing stop codons and the XhoI restriction site. The PCR products were purified and digested with the restriction enzyme XhoI. 3.5 μg of DNA was applied per well. The WT sequence does not have the XhoI site and wasn’t digested. On the other hand, the mutant sequences edited were cleaved by the XhoI restriction enzyme and produced two fragments, one with 216 bp and another with 941 bp. (**C)** Western blotting shows the deletion of the PGFS gene after CRISPR and also the expression of PGFS in the add-back parasites. The western blots are performed using polyclonal anti-PGFS antibody and monoclonal anti-α-tubulin as a normaliser. The fold-change was calculated using the WT parasites as standards. WT, wild-type; c27 and c29, mutant clones; MW, molecular weight; CN, negative control; bp, base pair; KDa, kiloDalton.

To generate PGFS knockout cell lines using CRISPR, in vitro transcribed sgRNA187 was used into multiple rounds of transfection of Cas9 expressing epimastigotes together with a donor DNA fragment (S1 Fig). Fig 2 shows the evaluation of ΔPGFS mutants obtained after the 4^th^ transfection with sgRNA187. The PGFS sequences were amplified using PCR, and the digestion profiles of the amplicons using the XhoI enzyme were analyzed by agarose gel electrophoresis. WT PGFS sequences have no recognition site for XhoI enzyme and therefore were not cleaved. The mutants have a site for XhoI, which was incorporated together with the stop codons during homologous recombination repair of the double strand DNA break (Fig 2B). Despite one mismatch between the guide RNA and one of the PGFS pseudogenes (S1 Fig), the results of the XhoI digestion analyses indicated that all PGFS sequences were cleaved by Cas9 and disrupted by the insertion of the donor DNA sequence.

The expression of the PGFS enzyme was evaluated by western blotting using a rabbit polyclonal anti-TcPGFS antibody [20] and anti-α-tubulin antibody, used for normalization. Fig 2C showed that even using large amounts of total proteins per well (140 μg), no PGFS expression was detected in two mutant cloned cell lines, further confirming that all copies were successfully disrupted. Fig 2C also shows that the add-back parasites effectively express PGFS after pROCK-HYG-PGFS transfection.

The growth of epimastigote forms of the WT parasites, parasites expressing the Cas9 and ΔPGFS mutant clones C27 and C29 was monitored by counting the parasites every 24 h. Given that there was no difference in parasite growth between WT and mutant parasites, our results demonstrated that PGFS is not an essential gene for epimastigotes (S3 Fig).

### The PGFS deletion does not change the susceptibility of the parasites to BZ and NFX

**Fig 3:**
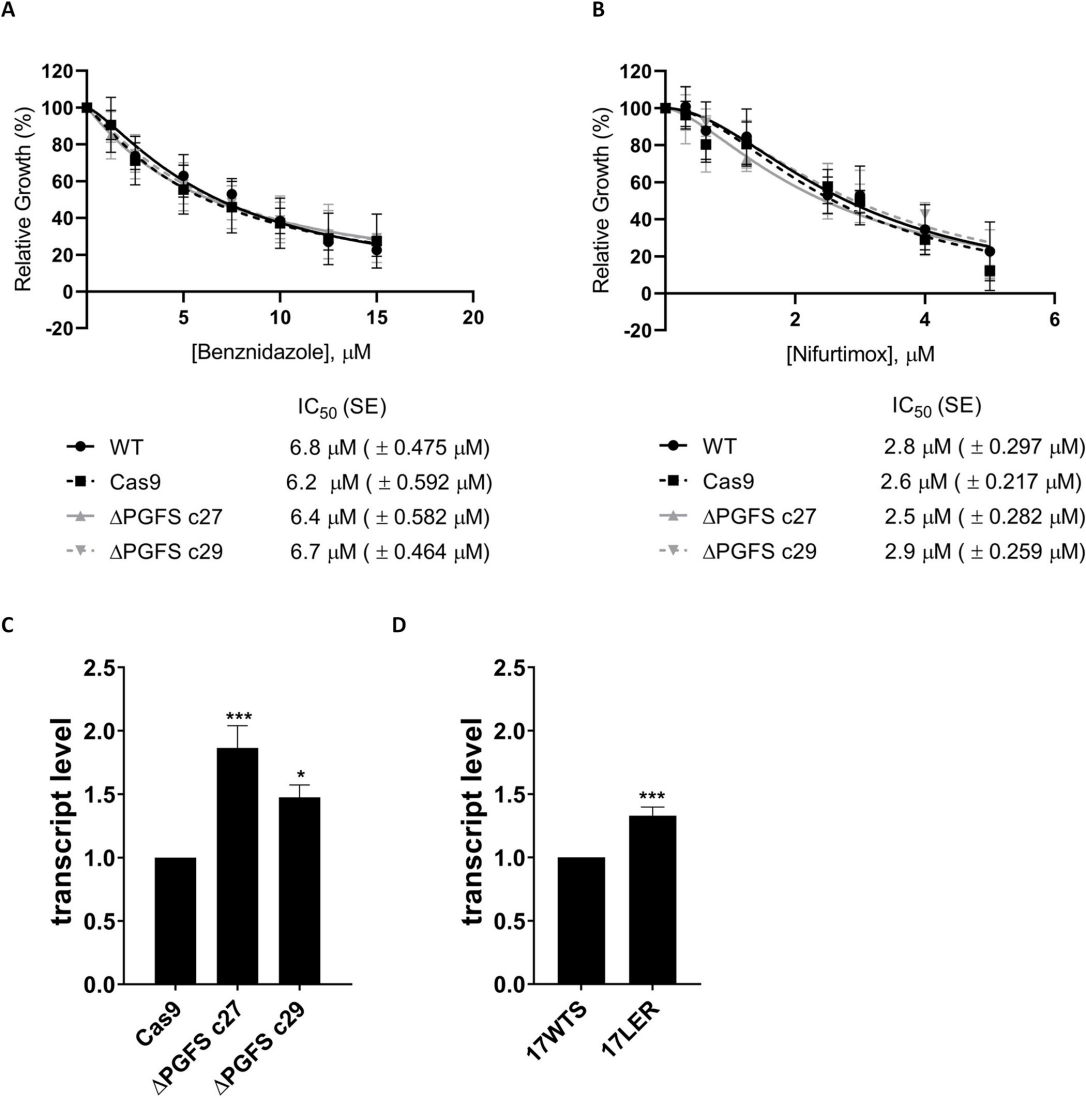
Drug susceptibility of ΔPGFS mutant parasites. Parasites were cultured in the presence of different concentrations of **(A)** benznidazole (1.25 to 15 μM) and **(B)** nifurtimox (0.3125 to 5 μM). Their growth was determined after 7 days of incubation with or without the drugs. Data represent the mean with standard deviations of three independent experiments performed in triplicate. The IC_50_ was determined through the nonlinear regression—variable slope model, using the "log (inhibitor) vs. response" equation in GraphPad Prism v.8.2.0. A Two-way ANOVA test with Bonferroni post hoc test was used to compare WT parasites and mutants for each drug concentration. * represents significant differences between the WT and the ΔPGFS clone c27 (* *p* < 0.05; ** *p* < 0.01; *** *p* < 0.001; **** *p* ≤ 0.0001). ^+^ represents significant differences between the WT and the ΔPGFS clone c29 (^+^ *p* < 0.05; ^++^ *p* < 0.01; ^+++^ *p* < 0.001; ^++++^ *p* ≤ 0.0001). ^#^ represents significant differences between the WT and the parasites expressing Cas9 (^#^ *p* < 0.05; ^##^ *p* < 0.01; ^###^ *p* < 0.001; ^####^ *p* ≤ 0.0001). **(C)** Comparison of NTR-1 transcription levels between the control parasite (Cas9) and ΔPGFS mutants. **(D)** Comparison of NTR-1 transcription levels between sensitive parasites (17WTS) and parasites whose resistance to benznidazole was induced in vitro (17LER). The housekeeping gene hypoxanthine-guanine phosphoribosyltransferase (HGPRT), was used as a constitutive normalizer and the fold-change was calculated by the 2^–ΔΔCt^ method. An Ordinary one-way ANOVA test with a Bonferroni post hoc test was used to compare WT parasites and mutants. * represents significant differences in relation to the control parasite (Cas9 or 17WTS) (* *p* < 0.05; ** *p* < 0.01; *** *p* < 0.001; **** *p* ≤ 0.0001).

PGFS deletion does not interfere with resistance to BZ, since the IC_50_ of WT parasites, parasites expressing the Cas9, and ΔPGFS mutant clones varied from 6.2 to 6.8 μM (Fig 3A). Similarly, NFX susceptibility was not altered by PGFS deletion since the IC_50_ of WT parasites, parasites expressing the Cas9 and ΔPGFS mutant clones varied from 2.5 to 2.9 μM (Fig 3B). No statistically significant difference was observed between the WT parasites and the mutants in any of the tested concentrations for any of these drugs.

To investigate why PGFS mutant clones exhibit the same susceptibility profiles to BZ and NFX as WT parasites and parasites expressing the Cas9, we examine NTR-1 transcript levels, since this enzyme is responsible for drug activation in *T*. *cruzi* [12]. RT-qPCR analyses indicated that NTR-1 transcript levels were 86% and 47% higher in the ΔPGFS mutant clones C27 and C29 respectively than in the parasites expressing the Cas9 (Fig 3C). Similarly, BZ-resistant parasites with fewer PGFS copies (previously described by Murta et al. 2006 [20]) had greater levels of NTR1, with resistant parasites having 33% more transcripts than sensitive parasites (Fig 3D).

### PGFS knockout parasites are less tolerant to oxidative stress generated by menadione

**Fig 4:**
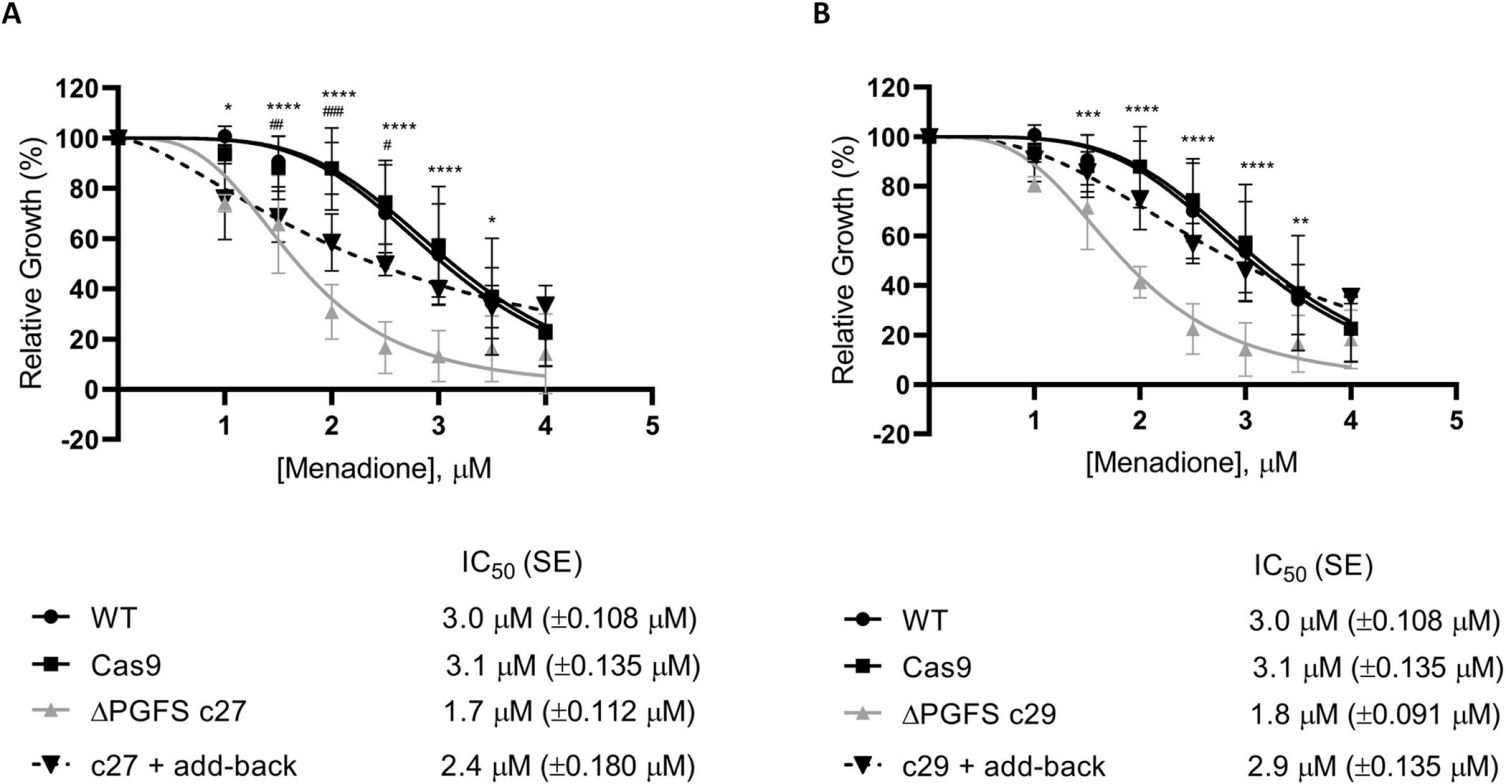
Tolerance of ΔPGFS mutant parasites to oxidative stress. Parasites were cultured in the presence of different concentrations of menadione (1.0 to 4 μM). Their growth was determined after 7 days of incubation with or without the drugs. **(A)** Comparison between the WT, control parasites expressing Cas9, ΔPGFS c27, and c27 + add-back parasites. **(B)** Comparison between the WT, control parasites expressing Cas9, ΔPGFS c29, and c29 + add-back parasites. Data represent the mean with standard deviations of three independent experiments performed in triplicate. The IC_50_ was determined through the nonlinear regression—variable slope model, using the "log (inhibitor) vs. response" equation in GraphPad Prism v.8.2.0. A Two-way ANOVA test with Bonferroni post hoc test was used to compare WT parasites and mutants for each drug concentration. * represents significant differences between the WT and the ΔPGFS clone c27, or between the WT and the ΔPGFS clone c29 (* *p* < 0.05; ** *p* < 0.01; *** *p* < 0.001; **** *p* ≤ 0.0001). ^#^ represents significant differences between the WT and the c27 + add-back, or between the WT and the c29 + add-back (^#^ *p* < 0.05; ^##^ *p* < 0.01; ^###^ *p* < 0.001; ^####^ *p* ≤ 0.0001).

To evaluate the effect of menadione-induced oxidative stress in the ΔPGFS mutant clones were incubated with different concentration of this drug. While the WT parasites and the Cas9-expressing parasites had an IC_50_ of 3.0 and 3.1 μM respectively, the ΔPGFS mutant clones C27 and C29 presented an IC_50_ of 1.8 and 1.9 μM respectively, which are 1.7 and 1.6-fold lower than the WT IC_50_ (Fig 4). The reintroduction of PGFS in the re-expressor clone ΔPGFS C29 completely recovered the phenotype of the parasites, and the re-expressor clone ΔPGFS C27 shows only a partially recovered phenotype. As shown in Fig 4, the IC_50_ of add-back for the clone C27 is 1.4 times greater than that of the knockout clone, whereas the IC_50_ add-back for the clone C29 is 1.6 times greater than that of the knockout (Fig 4).

### Knockout parasites have lower levels of lipid body vesicles in the stationary phase

**Fig 5:**
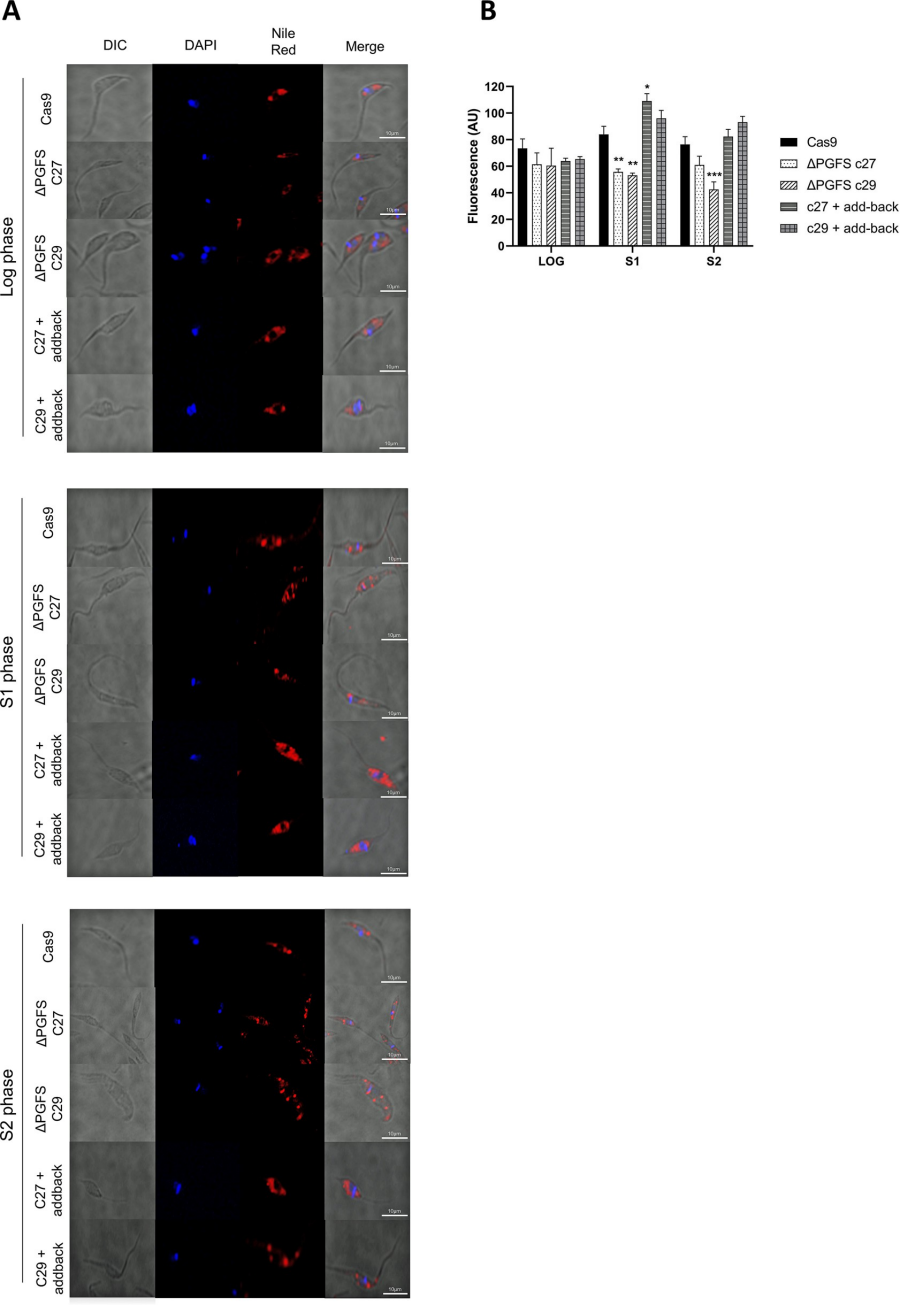
Evaluation of lipid body in ΔPGFS mutant parasites through Nile Red staining. The lipid droplets within the parasites were visualized and quantified using Nile Red reagent. **(A)** Representative images of the control parasites expressing Cas9, ΔPGFS clones, and add-back parasites at three distinct growth stages at three distinct growth stages: log, logarithmic phase (after 48 hours); s1, stationary phase 1 (after 7 days); s2 stationary phase 2 (after 15 days). **(B)** Geometric mean fluorescence in FL1 channel in arbitrary units comparing the quantity of lipid body vesicles in Cas9-expressing control parasites, PGFS clones, and add-back parasites at three distinct growth stages. A One-way ANOVA test with Bonferroni post hoc test was used to compare control parasites and mutant clones. *represents significant differences compared to the control parasite (Cas9) (* *p* < 0.05; ** *p* < 0.01; *** *p* < 0.001).

The metabolic transformation of arachidonic acid (AA) takes place in lipid bodies (LBs), and PGFS is present in this pathway. Although the parasites do not show differences in the amount of lipid body vesicles in the log phase, the stationary phase epimastigotes of the knockout parasites showed decreased levels of lipid body vesicles when compared to the control parasites. ΔPGFS clone C29 keeps presenting a significantly lower amount after two weeks of in vitro growth (Fig 5). On the other hand, the reintroduction of PGFS in the add-back parasites caused the levels of lipid bodies to become similar those of the control parasites.

### The absence of PGFS make parasites less infective

**Fig 6:**
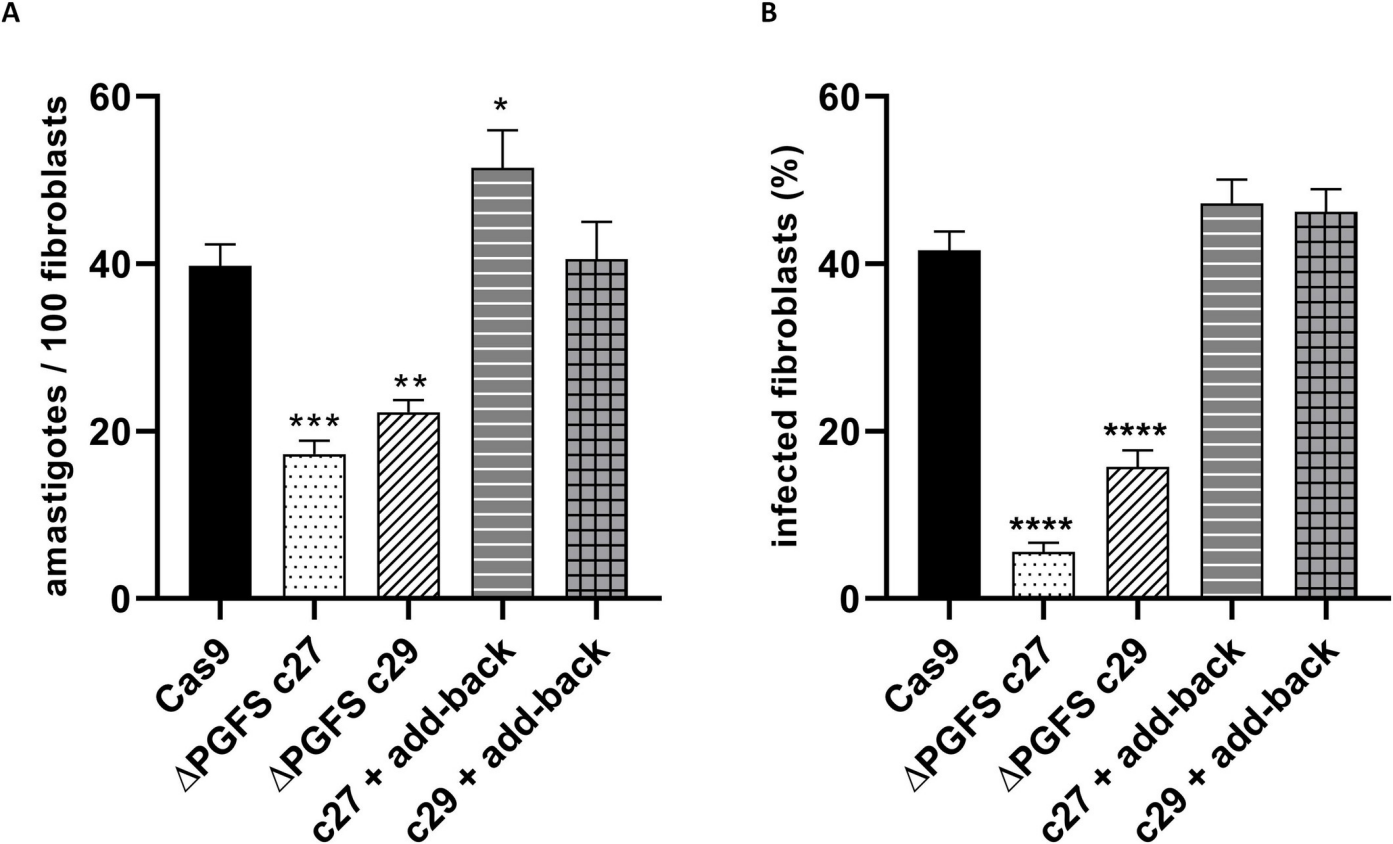
Infectivity ΔPGFS mutant parasites. L929 fibroblasts were infected with trypomastigote forms of control parasites expressing Cas9, ΔPGFS clones, and add-back parasites at a ratio of 1:10. Data represent the mean with standard deviations of three independent experiments performed in sextuplicate. **(A)** The graph shows the number of intracellular amastigotes per 100 macrophages 48h after infection. **(B)** The graph shows the percentage of infected fibroblasts. A One-way ANOVA test with Bonferroni post hoc test was used to compare parasites expressing Cas9 and mutant clones. *represents significant differences compared to the control parasite (Cas9) (* *p* < 0.05; ** *p* < 0.01; *** *p* < 0.001).

Since PGFS knockout mutants are less tolerant to oxidative stress and have showed decreased levels of lipid body vesicles, we asked whether the PGFS disruption alters the infectivity of the parasites. After infecting L929 fibroblasts with similar numbers of tissue culture derived trypomastigotes from Cas9 control parasites and ΔPGFS mutant parasites, we observed that the mutants had a smaller number of intracellular amastigotes 48 h after infection when compared to control parasites that express Cas9. As expected, add back parasites recovered infectivity after PGFS re-expression (Fig 6).

## Discussion

Given the large number of PGFS gene copies present in the CL Brener clone, the knockout result obtained here could only be achieved by the use of the CRISPR/Cas9 methodology, which proved to be highly efficient, as described in other studies of gene function of multigene families [32,38,39]. The CL Brener clone, was chosen as a reference strain for the Genome Project because of its well characterized phenotype, such as infectivity for the mammalian host, capacity to differentiate in vitro, and susceptibility to chemotherapy agents used in Chagas disease [40,41]. It was later shown that CL Brener is a hybrid strain belonging to DTU TcVI, [42] whose genome was incompletely assembled on a chromosomal level, particularly regarding regions harbouring multigene families, as PGFS [43]. Thus, to assess the correct PGFS copy number in *T*. *cruzi* we used a CL Brener genome assembled using SMRT long-reads. This method improves proper assembly by preventing multiple copies of genes from being collapsed into fewer copies. By using PacBio SMRT strategy we identified 8 different sequences of PGFS in CL Brener and this analysis was essential for the successful deletion of all PGFS copies in this parasite by CRISPR. Our results show the intra-family sequence variability in PGFS and reinforce the importance of having the complete set of genes from the target strain prior to designing CRISPR guide RNAs. This is especially important for hybrid strains as CL Brener, which can have variant gene copies originated from both parental strains. This variation among the sequences can be interpreted as a parasite’s adaptive mechanism to different environments [44–46].

It has previously been suggested that one of the mechanisms that can impart resistance to BZ in *T*. *cruzi* correlates with the deletion of copies of the PGFS gene [20]. The parasites of the 17LER population, which present *in vitro* induced BZ-resistance, only have one copy of the PGFS gene, while all other strains tested in this study have at least two copies. This study has also shown that 17LER resistant parasites had lower levels of PGFS transcripts than BZ-sensitive parasites [20]. Subsequently, a proteomics study showed that PGFS is less expressed in BZ-resistant *T*. *cruzi* [21]. In addition, studies have shown that *T*. *cruzi* parasites overexpressing PGFS are more susceptible to BZ and NFX [16,23], which suggests that this enzyme concentration can influence the drug resistance levels in the parasites. Unexpectedly, here we demonstrated that the PGFS knockout does not affect the growth of epimastigote forms or the parasite’s susceptibility to BZ or NFX.

To investigate why the PGFS mutant clones had the same susceptibility profile to BZ and NFX as control parasites, we measured NTR-1 transcript levels, as this enzyme is responsible for drug activation in *T*. *cruzi* [12,14]. NTR-1 transcripts were upregulated in PGFS knockout mutants as well as in BZ-resistant parasites with fewer PGFS copies. Thus, our results suggest that an increase in NTR-1 may play a role in the parasite retaining its sensitivity to BZ and NFX even in the absence of PGFS. Despite this data, it is wise to note that post-transcriptional regulation is crucial for controlling gene expression in trypanosomatids. As a result, changes in the NTR-1 mRNA levels may not necessarily be predictive of changes in protein levels. Remarkably, previous studies showed that ΔNTR-1 mutant *T*. *cruzi* is more resistant to BZ and NFX [12], suggesting that PGFS cannot compensate for the absence of NTR-1. Another study found that, among 5 different *T*. *cruzi* populations with resistance induced *in vitro* to BZ, one showed an increase in PGFS expression, concomitant with a decrease in NTR-1 levels, implying that PGFS is not capable of replacing NTR-1’s function in drug activation [14]. We cannot, however, rule out the possibility that genes other than NTR-1, whose expression levels were not examined, are also involved in the ΔPGFS phenotype.

Some other findings also indicates that PGFS may play a supporting role in drug resistance. A study revealed, for example, that the enzyme aldo-keto reductase (AKR), previously thought to be responsible for activating trypanocidal drugs, is probably linked to resistance mechanisms by eliminating glyoxal [47]. Notably, in *T*. *brucei* and *Leishmania infantum*, AKR has prostaglandin F2α synthase activity, but not in *T*. *cruzi*, since this role is instead played by PGFS in this parasite [47]. In this way, PGFS would be related to drug resistance, as previously demonstrated [20,21] but it would not be its main role.

To further investigate the role of PGFS in *T*. *cruzi* we assessed the parasite’s susceptibility to menadione, which induces oxidative stress by increasing the levels of peroxide and superoxide radicals [48]. While low levels of oxidants are essential for cell signalling, high levels cause harm to the parasites, and therefore they developed a sophisticated antioxidant defence system. This system not only deals with oxidants produced by cellular metabolism, but it is also critical for parasite survival within host cells and drug resistance [49]. A previous study has shown that parasites that overexpress PGFS are more resistant to H_2_O_2,_ and that WT parasites increase PGFS expression when exposed to H_2_O_2_ [16]_._ Here we have shown that the ΔPGFS mutants are more susceptible to oxidative stress than the WT parasites. These findings suggest that the main role of PGFS in parasite resistance mechanisms is likely to be linked to pathways such as defence against oxidative stress. In fact, the role of this enzyme in antioxidant defence has also been demonstrated in other organisms [50–53].

Lipid bodies (LBs), also known as lipid droplets, are highly active organelles involved in a wide range of biological processes. They contain lipids and proteins and are the site of the metabolic transformation of arachidonic acid (AA), a pathway in which PGFS is found [15–17]. As a result, as shown in the current study, a decrease in the expression of PGFS causes a reduction in the number of LBs in *T*. *cruzi* at some stages of growth.

Remarkably, the number of LBs found in protozoan parasites is linked to their virulence. It was shown, for example, that as *L*. *infantum* progresses to a virulent metacyclic stage, the number of LBs and the production of PGFS rise [54]. PGF_2_ is secreted by *T*. *cruzi* parasites, and its release has been linked to the parasites’ survival in the host, although its true function is yet unknown [15–17]. Previous studies have shown that, despite releasing a smaller amount of trypomastigotes *in vitro*, the parasites that overexpress PGFS present the peak of parasitemia in vivo six days before the peak of the WT parasites [16]. Furthermore, mice infected with the parasites that overexpress PGFS showed an increased parasite load in cardiac tissue [16]. Another study demonstrated that PGFS overexpression causes a decrease in the number of intracellular parasites when compared to controls [23]. Here we showed that ΔPGFS mutants have decreased infectivity and fewer lipid bodies than control parasites at some stages of growth. Briefly, we can state that PGFS is related to the infectivity of mutants, and we hypothesize that the divergent results obtained through deletion or overexpression may suggest a regulatory role for this enzyme.

## Supporting information

S1 Table PGFS genes in *T*. *cruzi*. (XLSX)

S2 Table PGFS genes in *T*. *cruzi* used in the phylogenetic analysis. (CSV)

S3 Table List of primers used in this study. (DOCX)

S1 Fig MAFFT alignment of the PGFS sequences in *T*. *cruzi* CL Brener. (DOCX)

S2 Fig PGFS translated sequences. (DOCX)

S3 Fig Growth of epimastigote forms of ΔPGFS mutant clones. An initial inoculum of 2 x 10^6^ parasites per mL was prepared for the WT parasites and clones 27 and 29, which were counted every 24 h using the Z1 Coulter Counter.
(TIF)
