# Supplementary material for: Disruption of multiple copies of the Prostaglandin F2alpha synthase gene affects oxidative stress response and infectivity in Trypanosoma cruzi
Source: PLoS Negl Trop Dis. 2022 Oct 19;16(10):e0010845. doi: 10.1371/journal.pntd.0010845 (PMC9581433; doi:10.1371/journal.pntd.0010845)
Supplement: S3 Table — (DOCX) [file pntd.0010845.s003.docx]

**Supporting information**

**S3 Table. List of primers used in this study.**

| **PRIMER NAME** | **PRIMER SEQUENCE 5’ → 3’** |
| --- | --- |
| sgRNA_PGFS_370_Fw | GGAGGCCGGAGAATTGTAATACGACTCACTATAGGGAGAGGGTCACTCTTCGACTGCTGAGTTTTAGAGCTAGAAATAGCAAG |
| Donor_PGFS_370 | AGCAAGGGGATCCTGGTCACTCTTCGACTGCTATCTATCTActcgagCTGAAGGATCTTCTCCGGAATCCCGGCTCG |
| sgRNA_PGFS_187_Fw | GGAGGCCGGAGAATTGTAATACGACTCACTATAGGGAGAGATGGTCCAGCCAAACTACACGTTTTAGAGCTAGAAATAGCAAG |
| Donor_PGFS_187 | GCCGAGGCGACGATGGTCCAGCCAAACTACTAGATAGATAGctcgagACTGGGTTTCTCACGGAGCCTGGCATTTAC |
| sgRNA_PGFS_351_Fw | GGAGGCCGGAGAATTGTAATACGACTCACTATAGGGAGAGAAGGATCTTCTCCGGAATCCGTTTTAGAGCTAGAAATAGCAAG |
| Donor_PGFS_351 | ACTCTTCGACTGCTGAAGGATCTTCTCCGGCTATCTATCTActcgagAATCCCGGCTCGACCAGCGTGAATGAGTTG |
| sgRNA_invitro_Rv | GGATCCAAAAAAGCACCGACTCGGTGCCAC |
| PGFS_Fw | ATGGCGACGTTCCCTGA |
| PGFS_Rv | TTATTTGTTGTACGTCGGGTAATC |
| RTqPCR_NTR1_Fw | CGGCAACGACGTTTCTTTATC |
| RTqPCR_NTR1_Rev | CACCGTGCCCAACACTAATA |
| RTqPCR_HGPRT_Fw | GGAGTACGAGTTTGCAGAGAAG |
| RTqPCR_HGPRT_Rev | GACCCTTTCCCTTGTAGTCATC |
| PGFS_pROCK_FW | TGCTCTATAAGTTGTCTTGTCTAGATGGCGACGTTCCCTGAAC |
| PGFS_pROCK_RV | gccttggagtcgtaaatggctcgagTTATTTGTTGTACGTCGGGTAATCG |
