# Supplementary material for: Disruption of multiple copies of the Prostaglandin F2alpha synthase gene affects oxidative stress response and infectivity in Trypanosoma cruzi
Source: PLoS Negl Trop Dis. 2022 Oct 19;16(10):e0010845. doi: 10.1371/journal.pntd.0010845 (PMC9581433; doi:10.1371/journal.pntd.0010845)
Supplement: S1 Fig — (DOCX) [file pntd.0010845.s004.docx]

**Supporting information**

**S1 Fig. MAFFT alignment of the PGFS sequences in *T. cruzi* CL Brener**

CLUSTAL format alignment by MAFFT (v7.408)

TcCLB.508461.80 atggcgacgttccctgaacttctgcggcccctcaaattggggcgctacacacttcgtaat

scaffold34_size atggcgacgttccctgaacttctgcggcccctcaaattggggcgctacacacttcgtaat

scaffold67_size atggcgacgttccctgaacttctgcggcccctcaaattggggcgctacacacttcgtaat

scaffold67_size atggcgacgttccctgaacttctgcggcccctcaaattggggcgctacacacttcgtaat

scaffold67_size atggcgacgttccctgaacttctgcggcccctcaaattggggcgctacacacttcgtaat

scaffold67_size atggcgacgttccctgaacttctgcggcccctcaaattggggcgctacacacttcgtaat

scaffold67_size atggcgacgttccctgaacttctgcgg-ccctcaaattggggcgctacacacttcgtaat

scaffold67_size atggcgacgttccctgaacttctgcggcccctcaaattggggcgctacacacttcgtaat

scaffold34_size atggcgacgttccctgaacttctgcggcccctcaaattggggcgctacacacttcgtaat

*************************** ********************************

TcCLB.508461.80 cggattattatggctcccttgacgcgttgccaggcaacagaagatggtcatgtaccaagg

scaffold34_size cggattattatggctcccttgacgcgttgccaggcaacagaagatgatcatgtaccaagg

scaffold67_size cggattattatggctcccttgacgcgttgccaggcaacagaagatggtcacgtaccaagg

scaffold67_size cggattattatggctcccttgacgcgttgccaggcaacagaagatggtcacgtaccaagg

scaffold67_size cggattattatggctcccttgacgcgttgccaggcaacagaagatggtcacgtaccaagg

scaffold67_size cggattattatggctcccttgacgcgttgccaggcaacagaagatggtcacgtaccaagg

scaffold67_size cggattattatggctcccttgacgcgttgccaggcaacagaagatggtcacgtaccaagg

scaffold67_size cggattattatggctcccttgacgcgttgccaggcaacagaagatggtcacgtaccaagg

scaffold34_size cggattattatggctcccttgacgcgttgccaggcaacagaagattgtcatgtaccaagg

********************************************* .***.*********

TcCLB.508461.80 acggaatcgatgctgaagtactacgaagaccgggcatctgcaggtcttatc-attgccga

scaffold34_size acggaatcgatgctgaagtactacgaagaccgggcatctgcaggtcttatc-attgccga

scaffold67_size acggaatcgatgctgaagtactacgaagaccgggcatctgcaggtcttatc-attgccga

scaffold67_size acggaatcgatgctgaagtactacgaagaccgggcatctgcaggtcttatc-attgccga

scaffold67_size acggaatcgatgctgaagtactacgaagaccgggcatctgcaggtcttatc-attgccga

scaffold67_size acggaatcgatgctgaagtactacgaagaccgggcatctgcaggtcttatc-attgccga

scaffold67_size acggaatcgatgctgaagtactacgaagaccgggcatctgcaggtcttatc-attgccga

scaffold67_size acggaatcgatgctgaagtactacgaagaccgggcatctgcaggtcttatcaattgccga

scaffold34_size acggaatcgatgctgaagtactacgaagaccgggcatctgcaggtcttatc-attgccga

*************************************************** ********

TcCLB.508461.80 ggcgacgatggtccagccaaacta-cactgggtttctcacggagcctggcatttactccg

scaffold34_size ggcgacgatggtccagccaaacta-cactgggtttctcacggagcctggcatttactccg

scaffold67_size ggcgacgatggtccagccaaacta-cactgggttcctcacggagcctggcatttactccg

scaffold67_size ggcgacgatggtccagccaaacta-cactgggttcctcacggagcctggcatttactccg

scaffold67_size ggcgacgatggtccagccaaacta-cactgggttcctcacggagcctggcatttactccg

scaffold67_size ggcgacgatggtccagccaaacta-cactgggttcctcacggagcctggcatttactccg

scaffold67_size ggcgacgatggtccagccaaacta-cactgggttcctcacggagcctggcatttactccg

scaffold67_size ggcgacgatggtccagccaaactaccactgggttcctcacggagcctggcatttactccg

scaffold34_size ggcgacgatggtccagccaaacta-cactgggtttctcacggagcctggcatttactccg

************************ *********.*************************

TcCLB.508461.80 atgcgcagattgaggagtggagaaagatcgtggacgcggtacacaaaaagggtggcctta

scaffold34_size atgcgcagattgaggagtggagaaagatcgtggacgcggtacacaaaaagggtggcctta

scaffold67_size atgcgcagattgaggagtggagaaagatcgtggacgcagtacacaaaaagggtggcctta

scaffold67_size atgcgcagattgaggagtggagaaagatcgtggacgcagtacacaaaaagggtggcctta

scaffold67_size atgcgcagattgaggagtggagaaagatcgtggacgcagtacacaaaaagggtggcctta

scaffold67_size atgcgcagattgaggagtggagaaagatcgtggacgcagtacacaaaaagggtggcctta

scaffold67_size atgcgcagattgaggagtggagaaagatcgtggacgcagtacacaaaaagggtggcctta

scaffold67_size atgcgcagattgaggagtggagaaagatcgtggacgcagtacacaaaaagggtggcctta

scaffold34_size atgcgcagattgaggagtggagaaagatcgtggacgcggtacacaaaaagggtggcctta

*************************************.**********************

TcCLB.508461.80 tattcctgcaactcattcacgctggtcgagccgggattccgga-gaagatccttcagcag

scaffold34_size tattcctgcaactcattcacgctggtcgagccgggattccgga-gaagatcctccagcag

scaffold67_size tattcctgcaactcattcacgctggtcgagccgggattccggg-gaagatccttcagcag

scaffold67_size tattcctgcaactcatccacgctggtcgagccgggattccggg-gaagatccttcagcag

scaffold67_size tattcctgcaactcatccacgctggtcgagccgggattccggg-gaagatccttcagcag

scaffold67_size tattcctgcaactcatccacgctggtcgagccgggattccggg-gaagatccttcagcag

scaffold67_size tattcctgcaactcatccacgctggtcgagccgggattccggg-gaagatccttcagcag

scaffold67_size tattcctgcaactcatccacgctggtcgagccgggattccggg-gaagatccttcagcag

scaffold34_size tattcctgcaactcattcacgctggtcgagccgggattccgggagaagatccttcagcag

****************.*************************. *********.******

TcCLB.508461.80 tcgaagagtgaccaggatccccttgctgggcgcctgcttgccccgagtgccattcccatt

scaffold34_size tcgaagagtgaccaggatccccttgctgggcgcctgcttgccccgagtgccattcccatt

scaffold67_size ccgaagagtgaccaggatccccttgctgggcgcctgcttgccgcgagtgccattcccatt

scaffold67_size ccgaagagtgaccaggatccccttgctgggcgcctgcttgccgcgagtgccattcccatt

scaffold67_size ccgaagagtgaccaggatccccttgctgggcgcctgcttgccgcgagtgccattcccatt

scaffold67_size ccgaagagtgaccaggatccccttgctgggcgcctgcttgccgcgagtgccattcccatt

scaffold67_size ccgaagagtgaccaggatccccttgctgggcgcctgcttgccgcgagtgccattcccatt

scaffold67_size ccgaagagtgaccaggatccccttgctgggcgcctgcttgccgcgagtgccattcccatt

scaffold34_size tcgaagagtgaccaggatccccttgctgggcgcctgcttgccccgagtgccattcccatt

.***************************************** *****************

TcCLB.508461.80 aaggaccatcggattcctgcctattttgctgcgagcggagaaaaggagacctacggtgtc

scaffold34_size aaggaccatcggattcctgcctattttgctgcgagcggagaaaaggagacctacggtgtc

scaffold67_size aaggaccatcggattcctgtctattttgctgcgagcggagaaaaggagacctacggtgtc

scaffold67_size aaggaccatcggattcctgtctattttgctgcgagcggagaaaaggagacctacggtgtc

scaffold67_size aaggaccatcggattcctgtctattttgctgcgagcggagaaaaggagacctacggtgtc

scaffold67_size aaggaccatcggattcctgtctattttgctgcgagcggagaaaaggagacctacggtgtc

scaffold67_size aaggaccatcggattcctgtctattttgctgcgagcggagaaaaggagacctacggtgtc

scaffold67_size aaggaccatcggattcctgtctattttgctgcgagcggagaaaaggagacctacggtgtc

scaffold34_size aaggaccatcggattcctgcctattttgctgcgagcggagaaaaggagacctacggtgtc

*******************.****************************************

TcCLB.508461.80 ccagaggagctcacggatgacgaagtccgggacggtatcatcccattgtttgtggagggg

scaffold34_size ccagaggagctcacggatgacgaagtccggaacggtatcatcccattgtttgtggagggg

scaffold67_size ccagaggagctcacggatgacgaagtccggaacggtatcataccattgtttgtggagggg

scaffold67_size ccagaggagctcacggatgacgaagtccggaacggtatcataccattgtttgtggagggg

scaffold67_size ccagaggagctcacggatgacgaagtccggaacggtatcataccattgtttgtggagggg

scaffold67_size ccagaggagctcacggatgacgaagtccggaacggtatcataccattgtttgtggagggg

scaffold67_size ccagaggagctcacggatgacgaagtccggaacggtatcataccattgtttgtggagggg

scaffold67_size ccagaggagctcacggatgacgaagtccggaacggtatcataccattgtttgtggagggg

scaffold34_size ccagaggagctca-ggatgacgaagtccgggacggtatcatcccattgtttgtggagggg

************* ****************.********** ******************

TcCLB.508461.80 gccaaaaacgccatctttaaggctgggtttgatggcgttgagattcatggagccaacggc

scaffold34_size gccaaaaacgccatctttaaggctgggtttgatggcgttgagattcatggagccaacggc

scaffold67_size gccaaaaacgccatttttaaggctgggtttgatggcgttgagattcatggagccaacggc

scaffold67_size gccaaaaacgccatttttaaggctgggtttgatggcgttgagattcatggagccaacggc

scaffold67_size gccaaaaacgccatttttaaggctgggtttgatggcgttgagattcatggagccaacggc

scaffold67_size gccaaaaacgccatttttaaggctgggtttgatggcgttgagattcatggagccaacggc

scaffold67_size gccaaaaacgccatttttaaggctgggtttgatggcgttgagattcatggagccaacggc

scaffold67_size gcc-aaaacgccatttttaaggctgggtttgatggcgttgagattcatggagccaacggc

scaffold34_size gccaaaaacgccatctttaaggctggg-ttgatggcgttgagattcatggagccaacggc

*** **********.************ ********************************

TcCLB.508461.80 tacttactggacg-ccttttttcgcgaatcttccaacaagcgccagt-ccggtccgtacg

scaffold34_size tacttactggacg-ccttttttcgcgaatcttccaacaagcgtcagt-ccggtccgtacg

scaffold67_size tacttactggacg-ctttttttcgcgaatcttccaacaagcgccagt-ccggtccgtacg

scaffold67_size tacttactggacg-ctttttttcgcgaatcttccaacaagcgccagt-ccggtccgtacg

scaffold67_size tacttactggacg-ctttttttcgcgaatcttccaacaagcgccagt-ccggtccgtacg

scaffold67_size tacttactggacg-ctttttttcgcgaatcttccaacaagcgccagt-ccggtccgtacg

scaffold67_size tacttactggacg-ctttttttcgcgaatcttccaacaagcgccagt-ccggtccgtacg

scaffold67_size tacttac--------------ttacgaa---tccaacaagcgccagt-ccggtccgtacg

scaffold34_size tacttactggacgcctttttttcgcgaatcttccaacaagcgccagtcccggtccgtacg

******* *..**** ***********.**** ************

TcCLB.508461.80 ccggaacgaccatcgacacacgatgccaactcatctacgatgtcaccaaaagcgtctgcg

scaffold34_size ccggaacgaccatcgacacacgatgccaactcatctacgatgtcaccaaaagcgtctgcg

scaffold67_size ccggaacgaccatcgacacacgatgccaactcatctacgatgtcaccaaaagcgtctgcg

scaffold67_size ccggaacgaccatcgacacacgatgccaactcatctacgatgtcaccaaaagcgtctgcg

scaffold67_size ccggaacgaccatcgacacacgatgccaactcatctacgatgtcaccaaaagcgtctgcg

scaffold67_size ccggaacgaccatcgacacacgatgccaactcatctacgatgtcaccaaaagcgtctgcg

scaffold67_size ccggaacgaccatcgacacacgatgccaactcatctacgatgtcaccaaaagcgtctgcg

scaffold67_size ccggaacgaccatcgacacacgatgccaactcatctacgatgtcaccaaaagcgtctgcg

scaffold34_size ccggaacgaccatcgacacacgatgccaactcatctacgatgtcaccaaaagcgtctgcg

************************************************************

TcCLB.508461.80 atgccgtgggaagtgaccgtgtggggctccgcatatccccactaaatggcgtgcatggga

scaffold34_size atgccgtgggaagtgaccgtgtggggctccgcatatccccactaatcggcgtgcatggga

scaffold67_size atgccgtgggaagtgaccgcgtggggctccgcatctccccactaaacggcgtgcatggga

scaffold67_size atgccgtgggaagtgaccgcgtggggctccgcatttccccactaaacggcgtgcatggga

scaffold67_size atgccgtgggaagtgaccgcgtggggctccgcatttccccactaaacggcgtgcatggga

scaffold67_size atgccgtgggaagtgaccgcgtggggctccgcatttccccactaaacggcgtgcatggga

scaffold67_size atgccgtgggaagtgaccgcgtggggctccgcatttccccactaaacggcgtgcatggga

scaffold67_size atgccgtgggaagtgaccgcgtggggctccgcatttccccactaaacggcgtgcatggga

scaffold34_size atgccgtgggaagtgaccgtgt-gggctccgcatat-cccactaaacggcgtgcatggga

*******************.** *********** * ******** .*************

TcCLB.508461.80 tgattgactcgaacccggaggcactaaccaagcatctatgcaagaaaattgagccacttt

scaffold34_size tgattgactcgaacccggaggcactaaccaagcatctatgcaagaaaattgagccacttt

scaffold67_size tgattgactcgaacccggaggcactaaccaagcatctatgcaagaaaattgagccacttt

scaffold67_size tgattgactcgaacccggaggcactaaccaagcatctatgcaagaaaattgagccacttt

scaffold67_size tgattgactcgaacccggaggcactaaccaagcatctatgcaagaaaattgagccacttt

scaffold67_size tgattgactcgaacccggaggcactaaccaagcatctatgcaagaaaattgagccacttt

scaffold67_size tgattgactcgaacccggaggcactaaccaagcatctatgcaag-aaattgagccacttt

scaffold67_size tgattgactcgaacccggaggcactaaccaagcatctatgcaagaaaattgagccacttt

scaffold34_size tgattgactcgaacccggaggcactaaccaagcatctatgcaagaaaattgagccacttt

******************************************** ***************

TcCLB.508461.80 cgcttgcctatctgcattacttgcgcggcgacatggtcaaccagcagattggtgacgttg

scaffold34_size cgcttgcctatctgcattacttgcgcggcgacatggtcaaccagcagattggtgacgttg

scaffold67_size cgcttgcctatctgcattacttgcgcggcgacatggtcaacgagcagattggcgacgttg

scaffold67_size cgcttgcctatctgcattacttgcgcggcgacatggtcaacgagcagattggcgacgttg

scaffold67_size cgcttgcctatctgcattacttgcgcggcgacatggtcaacgagcagattggcgacgttg

scaffold67_size cgcttgcctatctgcattacttgcgcggcgacatggtcaacgagcagattggcgacgttg

scaffold67_size cgcttg-ctatctgcattacttgcgcggcgacatggtcaacgagcagattggcgacgttg

scaffold67_size cgcttgcctatctgcattacttgcgcggcgacatggtcaacgagcagattggcgacgttg

scaffold34_size cgcttgcctatctgcattacttgcgcggcgacatggtcaaccagcagattggtgacgttg

****** ********************************** **********.*******

TcCLB.508461.80 tggcgtgggttcgtggaagttacagcggtgtaaaaatatccaacttgcgctacgatttcg

scaffold34_size tggcgtgggttcgtggaagttacagcggtgtaaaaatatccaacttgcgctacgatttcg

scaffold67_size tggcgtgggttcgtggaagttacagcggtgtaaaaatatccaacttgcgctacgatttcg

scaffold67_size tggcgtgggttcgtggaagttacagcggtgtaaaaatatccaacttgcgctacgatttcg

scaffold67_size tggcgtgggttcgtggaagttacagcggtgtaaaaatatccaacttgcgctacgatttcg

scaffold67_size tggcgtgggttcgtggaagttacagcggtgtaaaaatatccaacttgcgctacgatttcg

scaffold67_size tggcgtgggttcgtggaagttacagcggtgtaaaaatatcc-acttgcgctacgatttcg

scaffold67_size tggcgtgggttcgtggaagttacagcggtgtaaaaatatccaacttgcgctacgatttcg

scaffold34_size tggcgtgggttcgtggaagttacagcggtgtaaaaatatccaacttgcgctacgatttcg

***************************************** ******************

TcCLB.508461.80 aagaggcagaccagcaaatacgggaaggaaaagtcgacgccgtggcttttggcgccaagt

scaffold34_size aagaggcagaccagcaaatacgggaaggaaaagtcgacgccgtggcttttggcgccaagt

scaffold67_size aagaggcagaccagcaaatacgggaaggaaaagtcgacgccgtggcttttggcgccaagt

scaffold67_size aagaggcagaccagcaaatacgggaaggaaaagtcgacgccgtggcttttggcgccaagt

scaffold67_size aagaggcagaccagcaaatacgggaaggaaaagtcgacgccgtggcttttggcgccaagt

scaffold67_size aagaggcagaccagcaaatacgggaaggaaaagtcgacgccgtggcttttggcgccaagt

scaffold67_size aagaggcagaccagcaaatacgggaaggaaaagtcgacgccgtggcttttggcgccaagt

scaffold67_size aagaggcagaccagcaaatacgggaaggaaaagtcgacgccgtggcttttggcgccaagt

scaffold34_size aagaggcagaccagcaaatacgggaaggaaaagtcgacgccgtggcttttggcgccaagt

************************************************************

TcCLB.508461.80 tcattgcgaaccccgatctcgttgaaagggcccaacacaactggcccctcaacgagccgc

scaffold34_size tcattgcgaaccccgatctcgttgaaagggcccaacacaactggcccctcaacgagccgc

scaffold67_size tcattgcgaaccccgatctcgttgaaagggcccaacacaactggcccctcaacgagccgc

scaffold67_size tcattgcgaaccccgatctcgttgaaagggcccaacacaactggcccctcaacgagccgc

scaffold67_size tcattgcgaaccccgatctcgttgaaagggcccaacacaactggcccctcaacgagccgc

scaffold67_size tcattgcgaaccccgatctcgttgaaagggcccaacacaactggcccctcaacgagccgc

scaffold67_size tcattgcgaa-cccgatctcg-tgaaagggcccaacacaactggcccctcaacgagccgc

scaffold67_size tcattgcgaa-cccgatctcgttgaaagggcccaacacaactggcccctcaacgagccgc

scaffold34_size tcattgcgaaccccgatctcgttgaaagggcccaacacaactggcccctcaacgagccgc

********** ********** **************************************

TcCLB.508461.80 gaccagaaacatactacacaagaacagcagtcggatacaacgattacccgacgtacaaca

scaffold34_size gaccagaaacatacttcacaagaacagcagtcggatacaacgattacccgacgtacaaca

scaffold67_size gaccagaaacatactacacaagaacagcagtcggatacaacgattacccgacgtacaaca

scaffold67_size gaccagaaacatactacacaagaacagcagtcggatacaacgattacccgacgtacaaca

scaffold67_size gaccagaaacatactacacaagaacagcagtcggatacaacgattacccgacgtacaaca

scaffold67_size gaccagaaacatactacac-agaacagcagtcggatacaacgattacccgacgtacaaca

scaffold67_size gaccagaaacatactacacaagaacagcagtcggatacaacgattacccgacgtacaaca

scaffold67_size gaccagaaacatactacacaagaacagcagtcggatacaacgattacccgacgtacaaca

scaffold34_size gaccagaaacatactacacaagaacagcagtcggatacaacgattacccgacgtacaaca

*************** *** ****************************************

TcCLB.508461.80 aataa

scaffold34_size aataa

scaffold67_size a----

scaffold67_size a----

scaffold67_size a----

scaffold67_size a----

scaffold67_size a----

scaffold67_size a----

scaffold34_size aataa

*

sgRNA187 – yellow highlight
