# Supplementary material for: Disruption of multiple copies of the Prostaglandin F2alpha synthase gene affects oxidative stress response and infectivity in Trypanosoma cruzi
Source: PLoS Negl Trop Dis. 2022 Oct 19;16(10):e0010845. doi: 10.1371/journal.pntd.0010845 (PMC9581433; doi:10.1371/journal.pntd.0010845)
Supplement: S2 Fig — (DOCX) [file pntd.0010845.s005.docx]

**Supporting information**

**S2 Fig. PGFS translated sequences**

**Additional file 3: Figure S2.** PGFS translated sequences

>scaffold34_size588952_pilon:156143-157283(-)(GenBank:ON567257)

MATFPELLRPLKLGRYTLRNRIIMAPLTRCQATEDDHVPRTESMLKYYEDRASAGLIIAE

ATMVQPNYTGFLTEPGIYSDAQIEEWRKIVDAVHKKGGLIFLQLIHAGRAGIPEKILQQS

KSDQDPLAGRLLAPSAIPIKDHRIPAYFAASGEKETYGVPEELTDDEVRNGIIPLFVEGA

KNAIFKAGFDGVEIHGANGYLLDAFFRESSNKRQSGPYAGTTIDTRCQLIYDVTKSVCDA

VGSDRVGLRISPLIGVHGMIDSNPEALTKHLCKKIEPLSLAYLHYLRGDMVNQQIGDVVA

WVRGSYSGVKISNLRYDFEEADQQIREGKVDAVAFGAKFIANPDLVERAQHNWPLNEPRP

ETYFTRTAVGYNDYPTYNK-

>scaffold34_size588952_pilon:150143-151282(-)(GenBank:ON567258)

MATFPELLRPLKLGRYTLRNRIIMAPLTRCQATEDCHVPRTESMLKYYEDRASAGLIIAE

ATMVQPNYTGFLTEPGIYSDAQIEEWRKIVDAVHKKGGLIFLQLIHAGRAGIPGEDPSAV

EE-PGSPCWAPACPECHSH-GPSDSCLFCCERRKGDLRCPRGAQDDEVRDGIIPLFVEGA

KNAIFKAGLMALRFMEPTATYWTPFFRESSNKRQSRSVRRNDHRHTMPTHLRCHQKRLRC

RGK-PCGLRISH-TACMG-LTRTRRH-PSIYARKLSHFRLPICITCAATWSTSRLVTLWR

GFVEVTAV-KYPTCATISKRQTSKYGKEKSTPWLLAPSSLRTPISLKGPNTTGPSTSRDQ

KHTTQEQQSDTTITRRTTN

>scaffold67_size416336_pilon:174360-175496(+)(GenBank:ON567259)

MATFPELLRPLKLGRYTLRNRIIMAPLTRCQATEDGHVPRTESMLKYYEDRASAGLIIAE

ATMVQPNYTGFLTEPGIYSDAQIEEWRKIVDAVHKKGGLIFLQLIHAGRAGIPGKILQQP

KSDQDPLAGRLLAASAIPIKDHRIPVYFAASGEKETYGVPEELTDDEVRNGIIPLFVEGA

KNAIFKAGFDGVEIHGANGYLLDAFFRESSNKRQSGPYAGTTIDTRCQLIYDVTKSVCDA

VGSDRVGLRISPLNGVHGMIDSNPEALTKHLCKKIEPLSLAYLHYLRGDMVNEQIGDVVA

WVRGSYSGVKISNLRYDFEEADQQIREGKVDAVAFGAKFIANPDLVERAQHNWPLNEPRP

ETYYTRTAVGYNDYPTYN

>scaffold67_size416336_pilon:177540-178676(+) (GenBank:ON567260)

MATFPELLRPLKLGRYTLRNRIIMAPLTRCQATEDGHVPRTESMLKYYEDRASAGLIIAE

ATMVQPNYTGFLTEPGIYSDAQIEEWRKIVDAVHKKGGLIFLQLIHAGRAGIPGKILQQP

KSDQDPLAGRLLAASAIPIKDHRIPVYFAASGEKETYGVPEELTDDEVRNGIIPLFVEGA

KNAIFKAGFDGVEIHGANGYLLDAFFRESSNKRQSGPYAGTTIDTRCQLIYDVTKSVCDA

VGSDRVGLRISPLNGVHGMIDSNPEALTKHLCKKIEPLSLAYLHYLRGDMVNEQIGDVVA

WVRGSYSGVKISNLRYDFEEADQQIREGKVDAVAFGAKFIANPDLVERAQHNWPLNEPRP

ETYYTRTAVGYNDYPTYN

>scaffold67_size416336_pilon:190987-192123(+) (GenBank:ON567261)

MATFPELLRPLKLGRYTLRNRIIMAPLTRCQATEDGHVPRTESMLKYYEDRASAGLIIAE

ATMVQPNYTGFLTEPGIYSDAQIEEWRKIVDAVHKKGGLIFLQLIHAGRAGIPGKILQQP

KSDQDPLAGRLLAASAIPIKDHRIPVYFAASGEKETYGVPEELTDDEVRNGIIPLFVEGA

KNAIFKAGFDGVEIHGANGYLLDAFFRESSNKRQSGPYAGTTIDTRCQLIYDVTKSVCDA

VGSDRVGLRISPLNGVHGMIDSNPEALTKHLCKKIEPLSLAYLHYLRGDMVNEQIGDVVA

WVRGSYSGVKISNLRYDFEEADQQIREGKVDAVAFGAKFIANPDLVERAQHNWPLNEPRP

ETYYTRTAVGYNDYPTYN

>scaffold67_size416336_pilon:184643-185778(+) (GenBank:ON567262)

MATFPELLRPLKLGRYTLRNRIIMAPLTRCQATEDGHVPRTESMLKYYEDRASAGLIIAE

ATMVQPNYTGFLTEPGIYSDAQIEEWRKIVDAVHKKGGLIFLQLIHAGRAGIPGKILQQP

KSDQDPLAGRLLAASAIPIKDHRIPVYFAASGEKETYGVPEELTDDEVRNGIIPLFVEGA

KNAIFKAGFDGVEIHGANGYLLDAFFRESSNKRQSGPYAGTTIDTRCQLIYDVTKSVCDA

VGSDRVGLRISPLNGVHGMIDSNPEALTKHLCKKIEPLSLAYLHYLRGDMVNEQIGDVVA

WVRGSYSGVKISNLRYDFEEADQQIREGKVDAVAFGAKFIANPDLVERAQHNWPLNEPRP

ETYYTEQQSDTTITRRTT

>scaffold67_size416336_pilon:187816-188946(+) (GenBank:ON567263)

MATFPELLRPSNWGATHFVIGLLWLP-RVARQQKMVTYQGRNRC-STTKTGHLQVLSLPR

RRWSSQTTLGSSRSLAFTPMRRLRSGERSWTQYTKRVALYSCNSSTLVEPGFRGRSFSSR

RVTRIPLLGACLPRVPFPLRTIGFLSILLRAEKRRPTVSQRSSRMTKSGTVSYHCLWRGP

KTPFLRLGLMALRFMEPTATYWTLFFANLPTSASPVRTPERPSTHDANSSTMSPKASAMP

WEVTAWGSAFPH-TACMG-LTRTRRH-PSIYARN-ATFACYLHYLRGDMVNEQIGDVVAW

VRGSYSGVKISTCATISKRQTSKYGKEKSTPWLLAPSSLRTRSRERAQHNWPLNEPRPET

YYTRTAVGYNDYPTYN

>scaffold67_size416336_pilon:180716-181836(+) (GenBank:ON567264)

MATFPELLRPLKLGRYTLRNRIIMAPLTRCQATEDGHVPRTESMLKYYEDRASAGLINCR

GDDGPAKLPLGSSRSLAFTPMRRLRSGERSWTQYTKRVALYSCNSSTLVEPGFRGRSFSS

RRVTRIPLLGACLPRVPFPLRTIGFLSILLRAEKRRPTVSQRSSRMTKSGTVSYHCLWRG

PKRHF-GWV-WR-DSWSQRLLTYESNKRQSGPYAGTTIDTRCQLIYDVTKSVCDAVGSDR

VGLRISPLNGVHGMIDSNPEALTKHLCKKIEPLSLAYLHYLRGDMVNEQIGDVVAWVRGS

YSGVKISNLRYDFEEADQQIREGKVDAVAFGAKFIANPISLKGPNTTGPSTSRDQKHTTQ

EQQSDTTITRRTT
