# Supplementary figures and images for: Disruption of multiple copies of the Prostaglandin F2alpha synthase gene affects oxidative stress response and infectivity in Trypanosoma cruzi
Source: PLoS Negl Trop Dis. 2022 Oct 19;16(10):e0010845. doi: 10.1371/journal.pntd.0010845 (PMC9581433; doi:10.1371/journal.pntd.0010845)

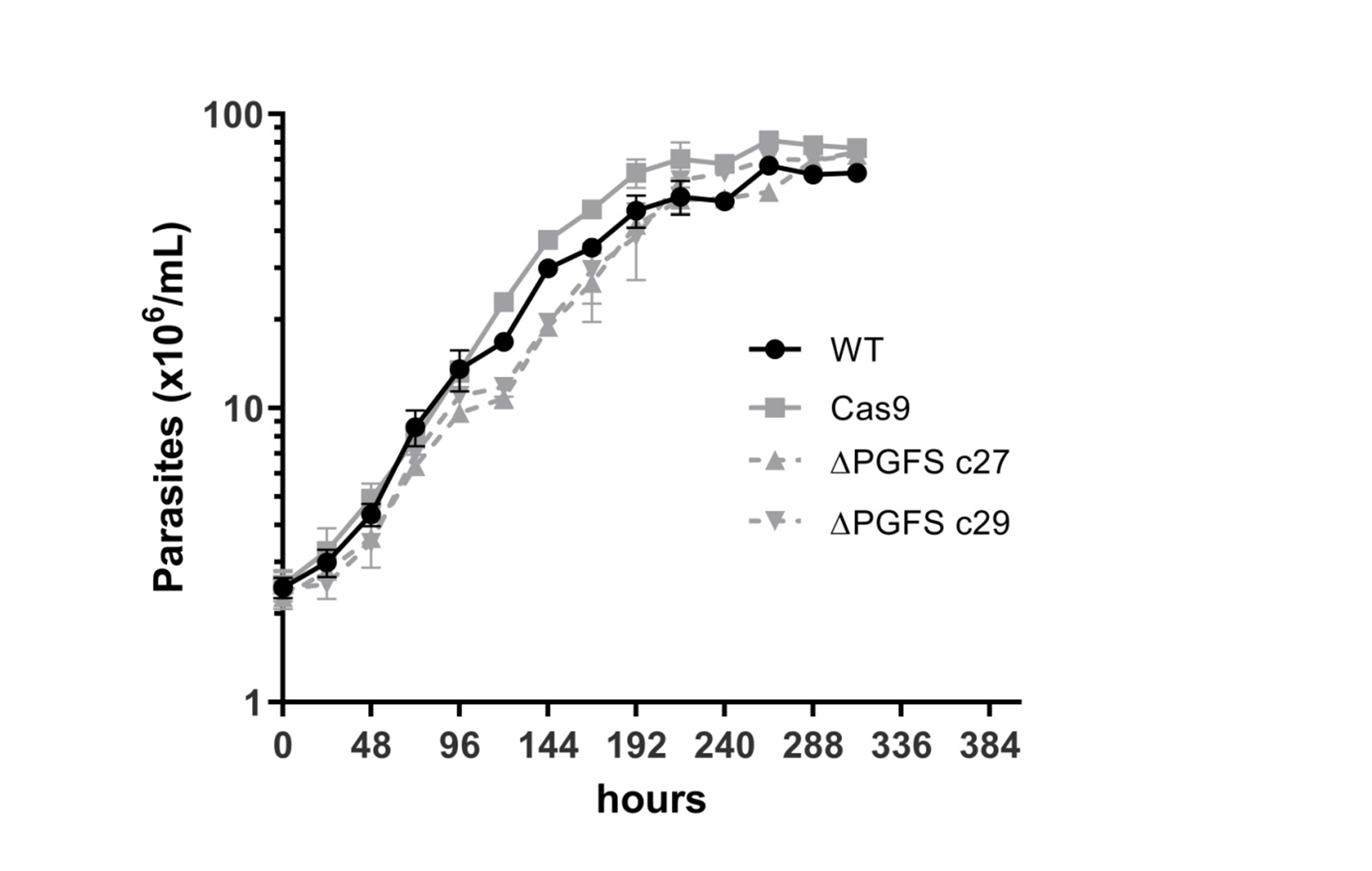

Supplement: S3 Fig — An initial inoculum of 2 x 10^6^ parasites per mL was prepared for the WT parasites and clones 27 and 29, which were counted every 24 h using the Z1 Coulter Counter. (TIF) [file pntd.0010845.s006.TIF]
